# Supplementary figures and images for: The overseas background of Chinese returnee energy scientists
Source: PLoS One. 2023 Nov 28;18(11):e0290959. doi: 10.1371/journal.pone.0290959 (PMC10684006; doi:10.1371/journal.pone.0290959)

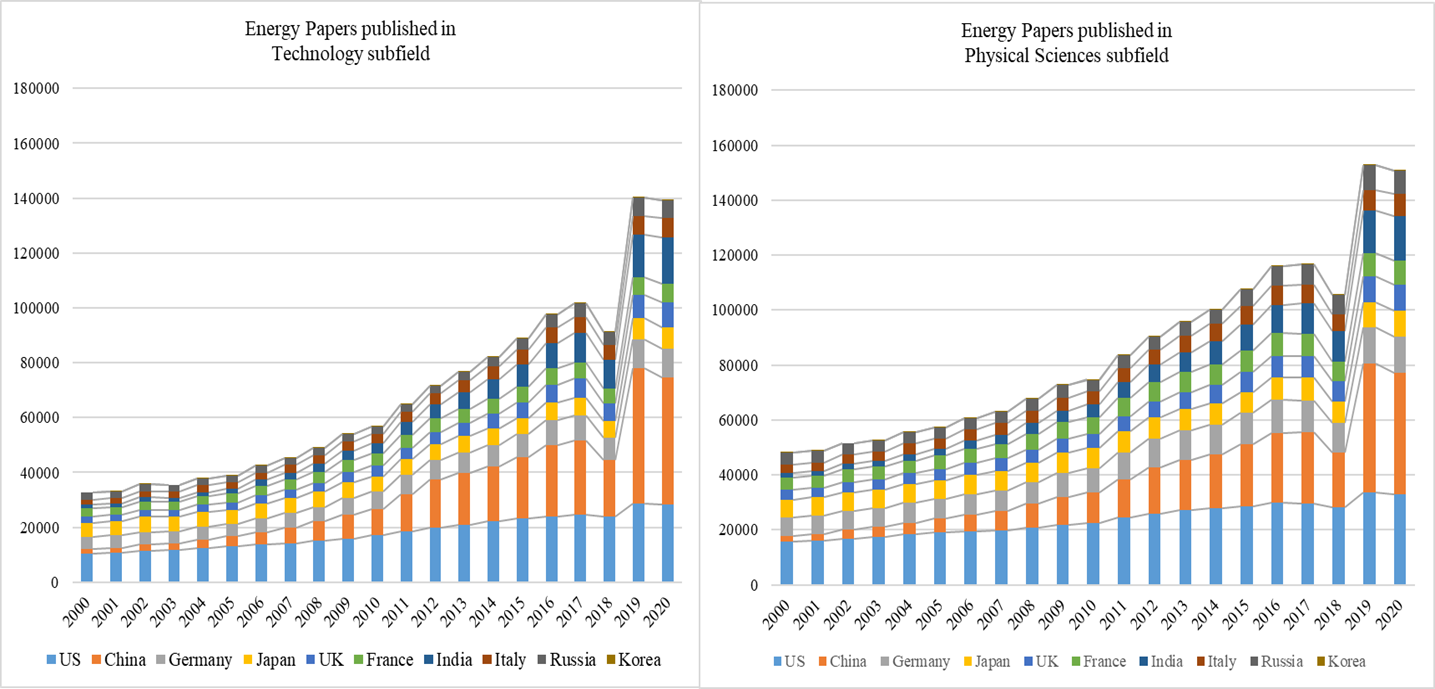

Supplement: S1 Fig — (TIF) [file pone.0290959.s001.tif]

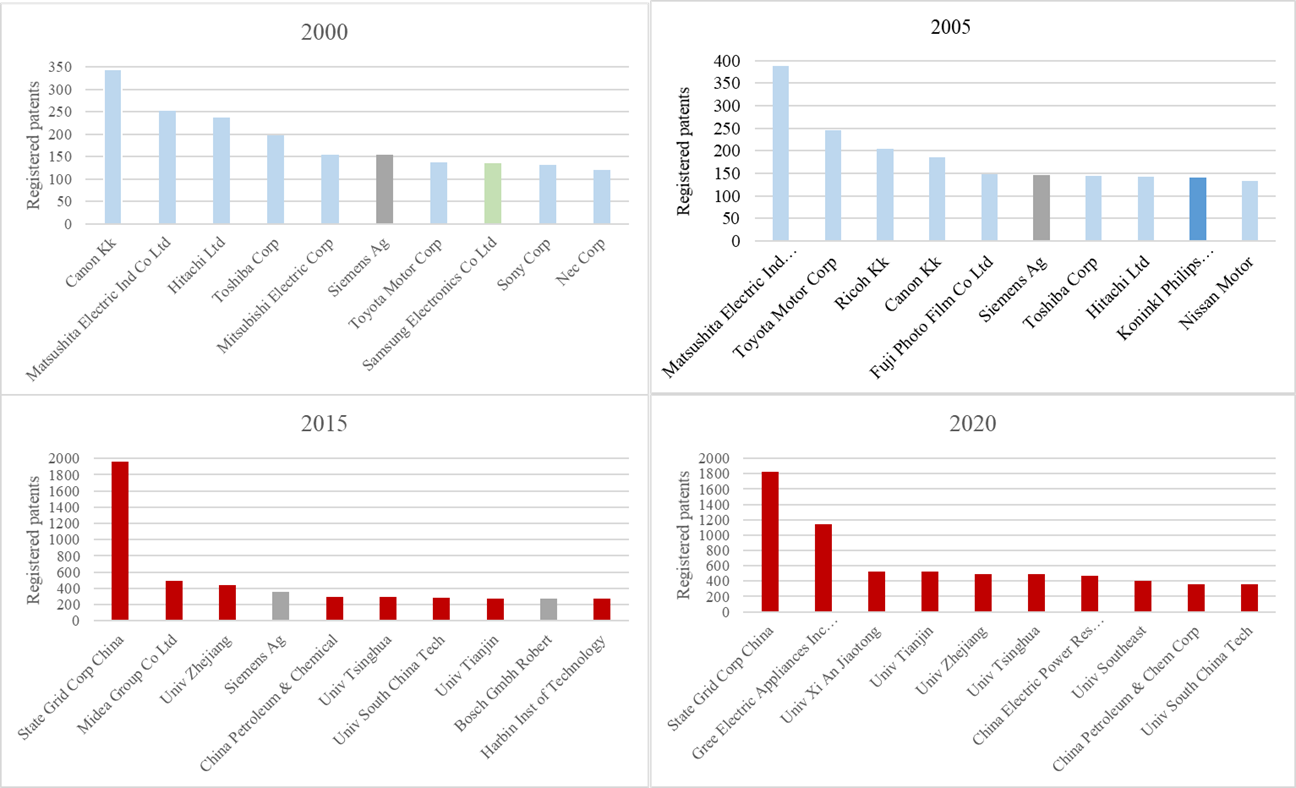

Supplement: S2 Fig — (TIF) [file pone.0290959.s002.tif]
