# Supplementary material for: The overseas background of Chinese returnee energy scientists
Source: PLoS One. 2023 Nov 28;18(11):e0290959. doi: 10.1371/journal.pone.0290959 (PMC10684006; doi:10.1371/journal.pone.0290959)
Supplement: Attachment — Submitted filename: PLOS ONE-Response to the editor-v3.docx [file pone.0290959.s004.docx]

July 5, 2023

Dear editors of PLOS ONE,

We appreciate your valuable feedback, and we have carefully revised the manuscript based on your suggestions. Below are our detailed responses to each suggestion:

1.“Please ensure that your manuscript meets PLOS ONE's style requirements, including those for file naming.”

**Our reply:** Thanks! Our manuscript format has been modified according to PLOS ONE style requirements, including use the correct file names.

2.“We note that you have indicated that data from this study are available upon request. PLOS only allows data to be available upon request if there are legal or ethical restrictions on sharing data publicly. For more information on unacceptable data access restrictions, please see <http://journals.plos.org/plosone/s/data-availability#loc-unacceptable-data-access-restrictions>.

In your revised cover letter, please address the following prompts:

a) If there are ethical or legal restrictions on sharing a de-identified data set, please explain them in detail (e.g., data contain potentially sensitive information, data are owned by a third-party organization, etc.) and who has imposed them (e.g., an ethics committee). Please also provide contact information for a data access committee, ethics committee, or other institutional body to which data requests may be sent.

b) If there are no restrictions, please upload the minimal anonymized data set necessary to replicate your study findings as either Supporting Information files or to a stable, public repository and provide us with the relevant URLs, DOIs, or accession numbers. For a list of acceptable repositories, please see <http://journals.plos.org/plosone/s/data-availability#loc-recommended-repositories>.”

**Our reply:** Thanks! In our revised cover letter, we explained that “In light of the potentially sensitive individual information contained in the original CV data of the energy scientists, we have decided not to publicly share the original data. However, interested parties can submit a reasonable data request to the corresponding author to gain access to the data. In addition, we have uploaded the aggregated and anonymized dataset that was used to generate all the figures and tables presented in our manuscript. The aggregated anonymized dataset is included as the Supporting Information Files.”

3.“Consider expand the description of both bibliographic databases of scientific (WoS) and patent publications (Derwent). Concerning WoS, expand the differentiation between disciplines, areas of research, and subject categories used (i.e., there are four subjects in energy science and technology, which were examined?).”

**Our reply:** Thanks for point out this concern. We also focused on the subfield of energy scientific paper publications within the realm of technology and physical sciences. The top ten countries in terms of scientific energy paper publications in these two subfields from 2000 to 2020 are presented in S1 Fig.

4.“Concerning patents, expand the patent search query and inclusion compare to other patent databases such as PATENTSCOPE or PATSTAT, does this refinement change your outlook?”

**Our reply:** Thanks for point out this concern. we also checked the top ten energy patentees in additional patent database: PATSTAT. The results stay consistent. Actually, search results from the PATSTAT suggest that Chinese patentees occupied eight positions in 2015 (compared to five in Derwent patent database), and all top ten energy patentees are Chinese entities in 2020 in the PATSTAT (compared to nine out of ten in Derwent patent database). The Top ten energy patentees from the PATSTAT database are reported in S2 Fig.

5.“Consider the accuracy of introducing the ‘big data CV’ term/concept. Since the sample is around ~1600 scientists, it is not clear why this sample should be considered as ‘big data.’”

**Our reply:** Thanks for point out this. The approximately 1600 energy scientists included in our study give a comprehensive representation of major research universities in China. We have taken note of your feedback and have removed any references or presentations related to "big data" from the manuscript.

6.“Consider expand the accuracy and completeness of institutional CV for researchers (e.g., out of ~1,600 resumes, how complete the information was retrieved? There was complete information for all variables (module, information, index, indicators) for all scientists?”

**Our reply:** Thanks for point out this. We revised the manuscript to explain the data collection process more clearly. “Following the CV collection, we engaged in a meticulous process of manually extracting all vital details. These details encompass the energy scientist's personal information, educational background, and postdoctoral work experience. Table 1 offers a snapshot of the kind of information extracted from the energy scientists' CVs. Any CVs found lacking in information were excluded from the research sample. Moreover, we manually supplemented the extracted data with each university's rank, as per the Quacquarelli Symonds University Ranking (QS Ranking). After eliminating CVs with incomplete data, we were left with a comprehensive set of 1,608 CVs energy scientists’ CVs. These CVs contained complete data for all the indicators specified in Table 1 and were retained for further analysis.”

So, for the 1,608 CVs we used to conduct all of our analysis, the information for all variables is complete.

7.“The article is presented in an intelligible fashion and is written in standard English. The article needs copy editing. There are missing words to make coherent sentences (e.g., lines 62-63: “This paper will [missing] China as an example […]; lines 70-71: “has a total of 2,078 articles, of which [missing] universities author 1,178”). The meaning of core ideas may be misleading given the current state of the paper.
There are sections in bold type for no particular reason (e.g., lines 131-132: “China has set ambitious goals, and improving energy efficiency is the key to achieving these goals”)”

**Our reply:** Thanks for point out this. We have diligently revised the manuscript, ensuring a coherent flow of information, removing unnecessary formatting such as bold displays, and enhancing the language for improved clarity and readability.

Sincerely,

Wenjing Lyu, Research Associate of Sloan School of Management,

Massachusetts Institute of Technology, Massachusetts, United States of America.

E-mail: [wjlyu@mit.edu](mailto:wjlyu@mit.edu)
